# Neutralizing the Th1 effector cytokines, IFN-γ and TNF-α, attenuates established experimental autoimmune anti-myeloperoxidase glomerulonephritis

**DOI:** 10.3389/fimmu.2025.1589130

**Published:** 2025-07-15

**Authors:** Kei Nagai, Daniel Koo Yuk Cheong, Anne Cao Le, Diana Shu Yee Tan, Joshua Daniel Ooi, Poh-Yi Gan

**Affiliations:** ^1^ Department of Medicine, Centre for Inflammatory Diseases, Monash University, Clayton, VIC, Australia; ^2^ Department of Nephrology, Division of Clinical Medicine, Faculty of Medicine, University of Tsukuba, Tsukuba, Japan

**Keywords:** autoimmune disease, ANCA-associated vasculitis, glomerulonephritis, antigen specific, T cells, anti-cytokine biologics

## Abstract

**Introduction:**

This study investigates the therapeutic potential of blocking key CD4^+^ Th1 effector cytokines, TNF-α and IFN-γ, in experimental anti-myeloperoxidase (MPO) glomerulonephritis (GN). The immunopathogenesis of MPO autoimmunity is biphasic, with an initial transient Th17 dominance followed by a sustained Th1 response, posing challenges for effective cytokine blockade.

**Methods:**

To evaluate anti-cytokine therapy at distinct disease phases, we induced anti-MPO autoimmunity in mice through MPO immunization and triggered GN using anti-glomerular basement membrane (GBM) globulin at early (day 20) and late (days 32 and 38) stages. Mice received anti-TNF-α or anti-IFN-γ beginning 4 hours post-GN induction, with kidney injury assessed 4 or 10 days later.

**Results:**

In early anti-MPO GN (day 20), neither anti-TNF-α nor anti-IFN-γ mitigated kidney injury, consistent with Th17-driven autoimmunity at this stage. However, in late, established GN (day 32), TNF-α blockade significantly attenuated kidney injury, indicating its pathogenic role in Th1-driven disease. At day 32, IFN-γ neutralization induced a Th2 phenotypic shift, increasing IL-4 production in *ex vivo* MPO-stimulated lymph node cells and upregulating alternatively activated M2 macrophages. Despite this immunological shift, short-term IFN-γ blockade failed to confer renal protection. To assess whether prolonged IFN-γ neutralization is beneficial, we extended the induced kidney injury phase to day 38. In this setting, extending anti-IFN-γ treatment effectively attenuated kidney injury, highlighting its therapeutic potential in late-stage disease.

**Conclusion:**

These findings highlight the dynamic role of Th1 cytokines in anti-MPO GN, with TNF-α blockade benefiting established disease, while IFN-γ neutralization requires prolonged intervention. This study informs Th1-targeted strategies in MPO anti-neutrophil cytoplasmic antibody (ANCA)-associated GN.

## Introduction

Myeloperoxidase-specific anti-neutrophil cytoplasmic antibody-associated glomerulonephritis (MPO-ANCA GN) is a significant cause of renal disease, particularly rapidly progressive GN. This autoimmune disease targets MPO, an enzyme sequestered in neutrophil azurophilic granules. Current treatments rely heavily on corticosteroids, which, while effective, cause considerable morbidity due to non-specific immunodepletion. Additional targeted therapeutic options like rituximab (depleting CD20 B cells) and avacopan (C5aR antagonist) are now utilized in clinical practice. Unlike other autoimmune conditions such as rheumatoid arthritis, systemic lupus erythematosus, and psoriasis, which have seen significant advances in incorporating emerging biological therapies, MPO-ANCA GN remains dependent on older non-specific treatments. The lack of advancement underscores the need for a deeper understanding of the disease’s underlying mechanisms, particularly the role of effector cytokines in MPO-ANCA GN. The immunopathogenesis of MPO-ANCA GN is increasingly recognized to involve CD4^+^ T cell subsets, including Th17 and Th1 cells. Peripheral blood mononuclear cells (PBMCs) from patients with MPO-ANCA GN produce IL-17A and IFN-γ in MPO recall responses, supporting the involvement of Th1 and Th17 responses (1). Recent spatial transcriptomic analyses of ANCA-GN patient kidney biopsies have revealed distinct glomerular and tubulointerstitial inflammatory niches marked by strong T cell activation signatures (2). Furthermore, single-cell transcriptomic profiling of kidney T cells identified dominant population of proinflammatory Th1 and Th17 effector cells in ANCA GN patients (2).

To study the underlying immunopathogenic mechanisms, we use an experimental model known as anti-MPO GN, in which mice are immunized with MPO in Freund’s adjuvant and GN is induced using a subnephritogenic anti-glomerular basement membrane (GBM) globulin. In this model, Th subset participation follows a biphasic pattern: early glomerular injury is dependent on Th17 cells, with GN effectively attenuated only during early disease in IL-23p19-deficient mice or following treatment with anti-IL-23p19 monoclonal antibody (mAb), whereas established GN relies on Th1-mediated inflammation and IL-12p35 (3). While anti-TNF-α mAbs have shown efficacy in experimental GN models at various stages of disease (4, 5), this study aimed to evaluate the therapeutic potential of targeting the key CD4^+^ Th1 signature cytokines, TNF-α and IFN-γ, in experimental anti-MPO GN by administering inhibitors at time points corresponding to the known Th17- and Th1-dominant phases of disease.

## Methods

### Mice

C57BL/6 (WT) mice were bred and housed in specific pathogen-free conditions at Monash Medical Centre (MMC) Animal Facilities, Monash University, Australia. Studies were approved by Monash University Animal Ethics Committee.

### Experimental design

*Early, developing experimental anti-MPO GN, day 20:* Mice were immunized subcutaneously (s.c.) with 20 µg recombinant murine MPO in Freund’s complete adjuvant (Sigma-Aldrich, Louis, MO) and boosted (day 7) with a further s.c. injection of 10 µg MPO in Freund’s incomplete adjuvant. GN was triggered by intravenous (i.v.) injection of 3 mg sheep anti-GBM globulin on day 16. Anti-MPO GN was assessed on day 20.

*Late, established experimental anti-MPO GN, days 32 and 38* (3): GN was triggered (day 28) in mice with induced anti-MPO autoimmunity (similar to day 20 protocol; MPO immunization, days 0 and 7), and experiments ended on day 32 to observe moderate glomerular disease. For extended induced glomerular injury, GN was induced on day 28 and kidney injury was assessed on day 38 to measure severe glomerular damage.

*In vivo cytokine blockade:* Anti-cytokine mAbs were administered intraperitoneally 4 h after GN induction and every other day. The following antibodies were used: hamster anti-mouse TNF-α mAb (250 µg/mouse; clone TN3.19.12, Bio X Cell) (6), rat anti-mouse IFN-γ mAb (500 µg/mouse; clone R4-6A2, Bio X Cell) (7), and rat IgG used as controls, matched accordingly by dose.

### Renal effector leukocyte—macrophage profiling

Kidney tissues were homogenized and digested in 4 mg/mL collagenase D (Roche Diagnostics, Indianapolis, IN) and 100 µg/mL DNase I in Hanks’ balanced salt solution without Ca^2+^ and Mg^2+^ (Sigma-Aldrich) for 30 min at 37°C on a rotor. Cells were erythrocytes lysed and samples filtered. Kidney cells were assessed using the following anti-mouse antibodies: anti-CD45 Pacific Blue (BioLegend), anti-IA/IE APC/Cy7 (BioLegend), anti-iNOS FITC (BD), and anti-CD206 PE (BioLegend). Voltages were optimized by single-color controls, and gates were drawn based on fluorescence minus one control. Data was collected on an LSRFortessa X-20 and analyzed with FlowJo.

### Assessment of renal disease and glomerular immune cell infiltration

Urine was collected by housing mice in individual metabolic cages over the final 24 h of the experiment. Albuminuria was assessed by ELISA (Bethyl Laboratories, Montgomery, TX) and expressed as µg/24 h. Urine creatinine were measured using standard methods at the MMC biochemistry laboratory. Histological assessment of renal injury was performed on 3-µm-thick, formalin-fixed, paraffin embedded, and periodic acid-Schiff stained kidney sections. A minimum of 30 glomeruli/mouse were examined, and results were expressed as the proportion of each glomerulus affected by segmental necrosis per glomerular cross-section (gcs). Glomerular CD4^+^ T cells, macrophages, and neutrophils were assessed by an immunoperoxidase staining technique on 6-µm-thick, PLP-fixed kidney sections. Primary antibodies were GK1.5 for CD4^+^ T cells (American Type Culture Collection, Manassas, VA), FA/11 for macrophages (anti-mouse CD68 from Dr. Gordon L Koch, Cambridge, England), and RB6-8C5 for neutrophils (anti-Gr-1, DNAX, Palo Alto, CA). A minimum of 30 glomeruli were assessed, and results were expressed as cells/gcs.

### Systemic autoimmune responses to MPO

ELISA was used to detect serum anti-MPO IgG, 1 µg/mL MPO, and horse radish peroxidase conjugated sheep anti-mouse IgG (Amersham Biosciences, Australia). To assess MPO-specific dermal delayed-type hypersensitivity (DTH), mice were challenged by intradermal injection of 10 µg/30 µL MPO in saline in the right footpad (contralateral footpad received saline). DTH was quantified 24 h later by measuring the difference between footpad thicknesses (Δmm) using a micrometer. For measurement of cytokine production of IFN-γ, IL-17A, TNF-α, and IL-4, draining lymph node cells were seeded at 2×10^6^ cells well cultured with 10 μg/mL of MPO for 72 h. Cytokines in supernatant were measured by ELISA (8, 9).

### Statistics

Data were analyzed with Graph Pad Version 6 (GraphPad Software Inc., San Diego, CA). Results are expressed as the mean ± SEM. Unpaired *t-*test was used when comparing two groups.

## Results

### Timing-dependent efficacy of anti-IFN-γ treatment in experimental anti-MPO glomerulonephritis

To determine if blocking IFN-γ can treat anti-MPO GN, anti-IFN-γ mAb was administered during the early Th17-dominant phase of anti-MPO GN (day 20). At this stage, anti-IFN-γ mAb treatment was unable to improve GN (glomerular segmental necrosis and albuminuria) or serum MPO-ANCA IgG (**Supplementary Figures S1A–C**). Compared to control treatment, MPO-specific dermal DTH swelling was significantly reduced in mice receiving anti-IFN-γ mAb (**Supplementary Figure S1D**). This was associated in a significant reduction in MPO-stimulated splenocyte production of IFN-γ; however, there was no difference in TNF-α or IL-17A production (**Supplementary Figures S1E–G**).

**Figure 1:**
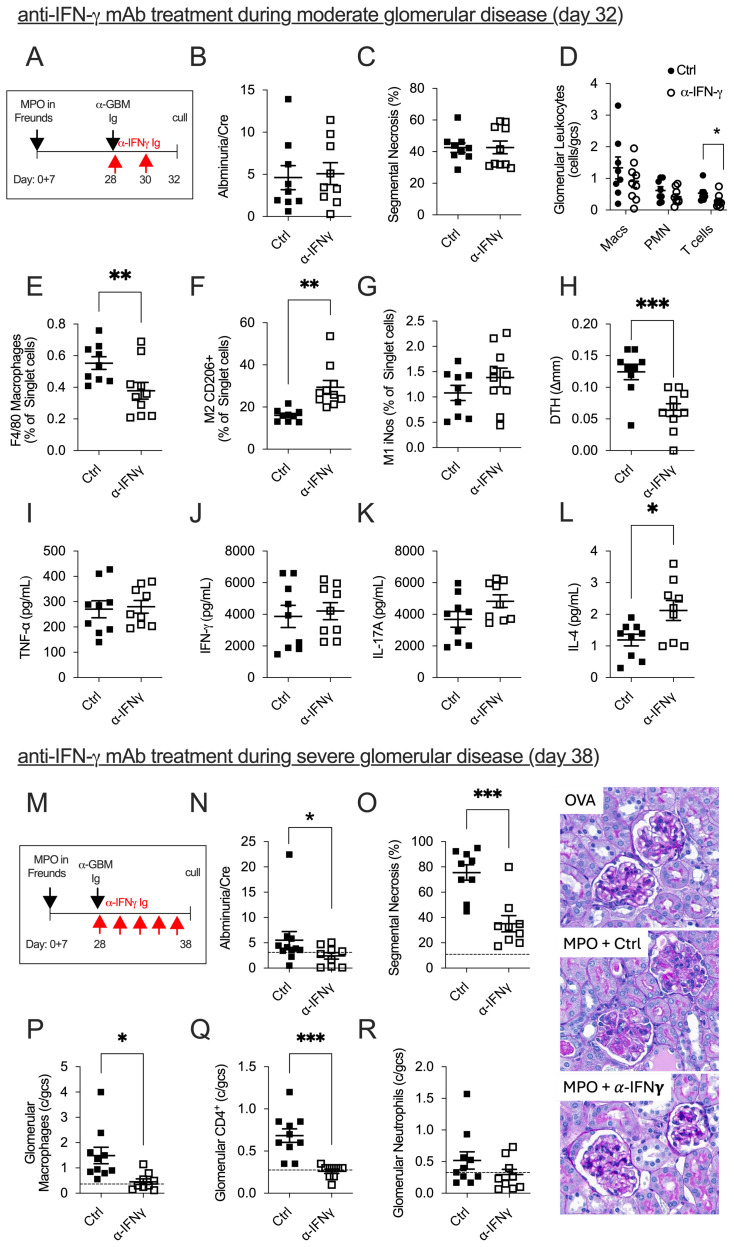
The efficacy of anti-IFN-γ mAb treatment in mice with established anti-MPO GN is dependent on disease severity and timing. In mice with established moderate anti-MPO GN **(A)**, anti-IFN-γ mAb treatment failed to improve kidney damage compared to control-treated mice **(B, C)**. However, there were signs of kidney improvement, including reductions in glomerular CD4^+^ T cells, and intrarenal macrophages **(D, E)**. In anti-IFN-γ mAb-treated mice, the frequency of protective M2 macrophages increased, while the frequency of injurious M1 macrophages remained unchanged **(F, G)**. Reductions in MPO-specific DTH footpad swelling were observed in mice treated with anti-IFN-γ mAb **(H)**. There was no difference in MPO-specific recall responses, including TNF-α, IFN-γ, or IL-17 production from splenocytes draining MPO immunization sites **(I–K)**. However, IFN-γ blockade modulated systemic anti-MPO immune responses by enhancing splenic IL-4 production **(L)**. In mice with severe anti-MPO GN, anti-IFN-γ mAb administered over 10 days successfully attenuated both functional (proteinuria) and structural (glomerular segmental necrosis, glomerular macrophage, glomerular CD4^+^ T cells) kidney damage compared to control-treated mice **(M–Q)**. No difference in glomerular neutrophils was observed between groups **(R)**. Dotted line represents control ovalbumin-immunized mice administered with anti-GBM globulin. *P<0.05, **P<0.01, ***P<0.0001.

To determine if anti-IFN-γ mAb treatment would be efficacious when Th1 cells predominate, i.e., mimicking conditions when patients are commonly diagnosed, we developed a modified model of anti-MPO GN, whereby disease is triggered in mice at day 28 when Th1 immune responses are well established. We also chose to measure disease 4 days post-trigger when disease is moderate and at 10 days when disease is severe (**Figure 1A**). At 4 days post-trigger, anti-IFN-γ mAb treatment was ineffective in attenuating glomerular injury. Compared to control-treated mice, anti-IFN-γ mAb made no difference in albuminuria (**Figure 1B**), glomerular segmental necrosis (**Figure 1C**), or glomerular neutrophils and glomerular macrophages (**Figure 1D**). However, a significant reduction in the influx of glomerular CD4^+^ T cells, intrarenal macrophages (CD45^+^F4/80^+^I-A/I-E^+^), and a shift toward renoprotective M2 macrophages (CD45^+^F4/80^+^I-A/I-E^+^Mannose Receptor^+^) from injurious M1 macrophages (CD45^+^F4/80^+^I-A/I-E^+^iNOS^+^) were observed (**Figures 1D–G**). Assessment of anti-MPO immune responses showed a significant reduction in anti-MPO DTH footpad swelling (**Figure 1H**) in mice receiving anti-IFN-γ mAb, but no difference in *in vitro* MPO-stimulated splenocyte production of TNF-α, IFN-γ, and IL-17A was observed (**Figures 1I–K**). While there was no change in the dominant Th1 and Th17 effector responses, there was a significant increase in the splenic production of the Th2-dominant effector cytokine, IL-4 (**Figure 1L**). This experiment demonstrates that during the 4-day effector phase of glomerular injury, anti-IFN-γ mAb treatment skewed Th1 responses to a Th2 response with evidence of increased anti-MPO specific splenic IL-4 production and increased frequency of anti-inflammatory renal M2 macrophages. However, no overall protection in GN was observed. Ten days after the induction of GN (**Figure 1M**), we found significantly improved kidney injury outcomes in mice given anti-IFN-γ mAb, with decreased proteinuria (**Figure 1N**) and attenuated severity of glomerular damage (glomerular segmental necrosis, glomerular macrophages, and glomerular CD4^+^ T cells, **Figures 1O–Q**). However, no difference was observed in the recruitment of glomerular neutrophils between groups (**Figure 1R**). Therefore, extending the treatment protocol of anti-IFN-γ mAb allows for successful modulation of injurious Th1 effector responses that significantly attenuate anti-MPO GN. These findings demonstrate that administering anti-IFN-γ therapy after anti-MPO autoimmunity is established can modulate Th1 responses and significantly reduce the severity of disease.

### Anti-TNF-α mAb treatment effectively treats mice with established anti-MPO GN (day 32)

Similar to findings using anti-IFN-γ mAb, administration of anti-TNF-α mAb during the early Th17-dominant phase of anti-MPO GN (day 20) failed to improve GN. Mice treated at this stage showed a comparable degree of glomerular segmental necrosis, albuminuria, and serum MPO-ANCA compared to controls (**Supplementary Figures S2A–C**). However, modulation of MPO-specific immune responses was evident, with a reduction in DTH footpad swelling in anti-TNF-α-treated mice (**Supplementary Figure S2D**). The effect of neutralizing TNF-α was confirmed by increased serum TNF-α levels (**Supplementary Figure S2E**), while the concentration of TNF-α following 72-h culture of splenocytes stimulated with MPO was significantly reduced (**Supplementary Figure S2F**). No differences were observed in the concentration of splenic production of IFN-γ and IL-17A (**Supplementary Figures S2 G–H**).

**Figure 2:**
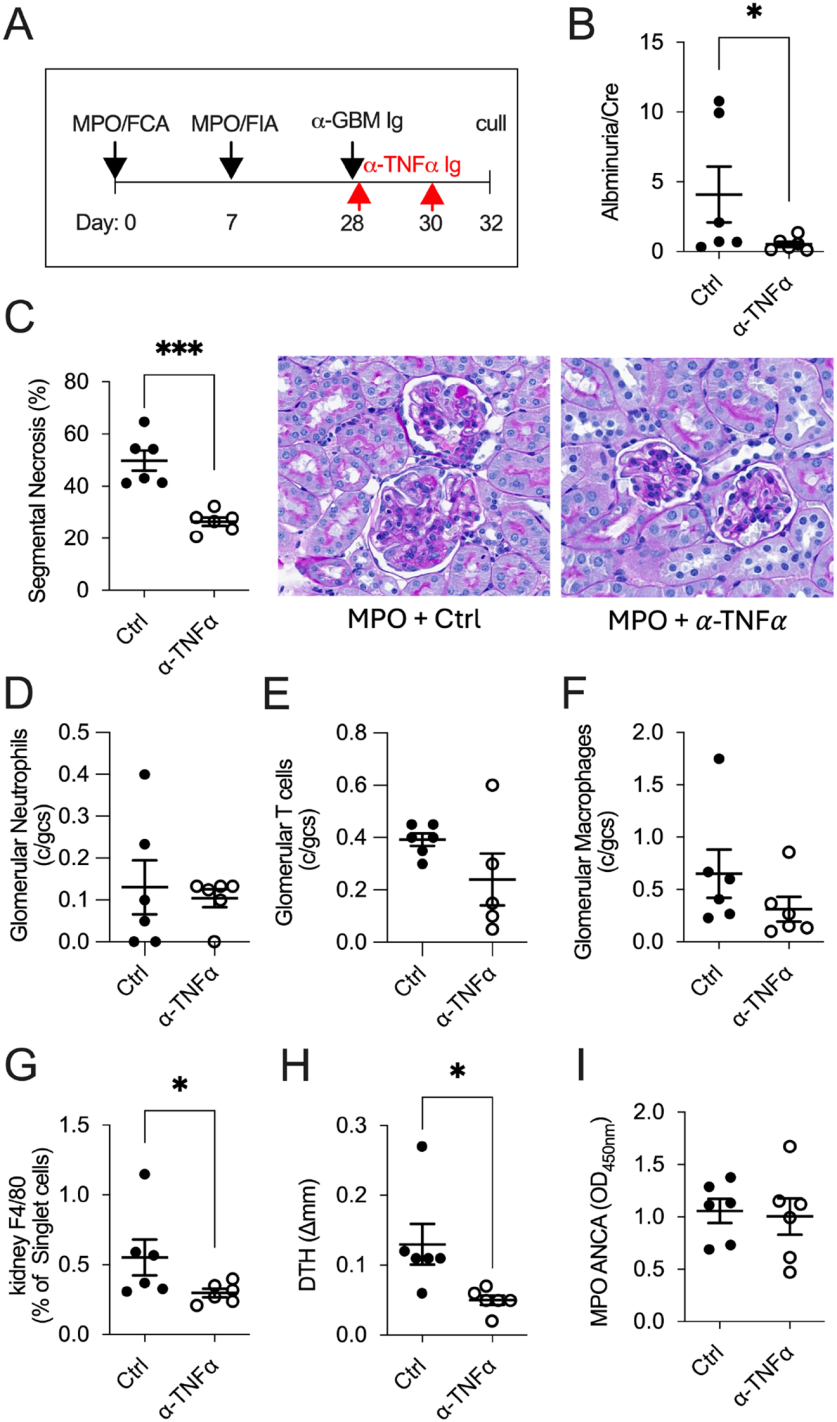
TNF-α blockade effectively treats mice in Th1-dominant anti-MPO GN disease (day 32). In mice with established anti-MPO GN **(A)**, treatment with anti-TNF-α mAb reduced kidney injury, as indicated by decreased albuminuria **(B)** and glomerular segmental necrosis **(C)** compared with control-treated mice. There was no reduction in glomerular leukocyte recruitment between groups **(D–F)**. Neutralizing TNF-α reduced intrarenal macrophages **(G)** and anti-MPO specific DTH swelling **(H)** but showed no differences in serum MPO-ANCA IgG titers **(I)**. *P<0.05, ***P<0.0001.

Neutralizing TNF-α when MPO-specific Th1 immune responses were established in anti-MPO GN (day 32; **Figure 2A**) significantly attenuated kidney damage compared to control-treated mice, assessed by albuminuria (**Figure 2B**) and glomerular segmental necrosis (**Figure 2C**). Although reductions in glomerular leukocyte recruitment in anti-TNF-α-treated mice did not reach statistical significance (**Figures 2D–F**), the improvement in glomerular injury was associated with reduced intrarenal macrophages (% F4/80^+^ of CD45^+^ cells) and reduced cellular-mediated anti-MPO DTH footpad swelling in anti-TNF-α-treated mice (**Figures 2G–H**). Serum MPO-ANCA IgG titers remained unchanged between groups (**Figure 2I**).

Collectively, these results demonstrate that biologicals targeting Th1 signature effector cytokines are effective at treating anti-MPO GN when immune responses are established.

## Discussion

IFN-γ and TNF-α are critical effector cytokines in inflammation, immune cell activation, and immunoregulation. TNF-α drives inflammation and tissue destruction, while IFN-γ promotes Th1-mediated cellular immunity. In autoimmune diseases, these cytokines create a pathogenic feed-forward loop between macrophages and Th1 cells. This loop perpetuates and amplifies the inflammatory response and has been demonstrated to contribute to the pathogenesis of MPO-ANCA GN, making these cytokines a potential therapeutic target.

The translation of TNF-α blockade (i.e., infliximab and etanercept) into a therapy for ANCA-associated GN (ANCA GN), including patients with granulomatosis with polyangiitis (GPA) (PR3-ANCA) and microscopic polyangiitis (MPO-ANCA), has faced challenges. An open-label, multi-center, prospective clinical trial showed 88% remission in severe ANCA GN patients treated with infliximab (10). However, the WEGET trial, a larger randomized, placebo-controlled study, found no significant difference in remission maintenance between etanercept and placebo in granulomatosis with polyangiitis patients with mild disease severity (11). Concerns about an increased incidence of solid cancers in etanercept-treated patients further complicated its use. Moreover, a randomized trial comparing infliximab and rituximab (anti-CD20 mAb) for refractory GPA patients found infliximab less effective (12). Overall, trials blocking TNF-α in ANCA GN have yielded mixed results, underscoring the need for improved patient stratification based on disease severity.

Our experimental model of anti-MPO GN closely mirrors the T helper cell dynamics observed in human disease, providing valuable insights into the role of T cell cytokine-driven mechanisms of injury (3). This is supported by a recent human study by Engesser et al., which used spatial and single-cell transcriptomic profiling in ANCA-associated vasculitis (AAV) patients to reveal distinct enrichment of intrarenal CD4^+^ Th1 and Th17 cells (2). Additionally, immunoprofiling of intrarenal T cells from four patients with established ANCA GN who did not respond to rituximab therapy demonstrated an increased accumulation of Th1 cells. Our murine model reflects this T-cell-dependent pathology (3), allowing us to dissect the roles of Th1 effector cytokines IFN-γ and TNF-α in established disease stages and assess therapeutic interventions relevant to human disease progression.

In contrast to our earlier work, Gan et al. (2019) (3), which focused on the upstream blockade of Th1 differentiation using anti-IL-12p35 or anti-IL-12/23p40, the current study investigates the therapeutic effects of targeting downstream Th1 effector cytokines, specifically TNF-α and IFN-γ. Importantly, this study also extends the effector phase of anti-MPO GN from 4 days to 10 days, allowing us to better evaluate the potential of these cytokine inhibitors in severe established disease.

Anti-TNF-α therapy administered at day 28 significantly reduced glomerular inflammation and intrarenal macrophage accumulation. This finding elucidates why the initial prospective clinical trial demonstrated high remission rates in more severe AAV patients; that is, TNF-α blockade is effective in reducing inflammation and tissue damage in advanced stages of the disease, highlighting the importance of disease severity and timing in therapeutic efficacy.

In contrast, IFN-γ blockade during severe anti-MPO GN demonstrated renoprotective effects, modulating disease by switching Th1-mediated anti-MPO autoimmune responses to Th2 responses and polarizing intrarenal macrophages into renoprotective M2 macrophages. However, IFN-γ blockade required sustained administration to achieve therapeutic benefit, suggesting slower kinetics compared to TNF-α inhibition. While no anti-IFN-γ therapies are currently FDA-approved for autoimmune diseases, early-phase clinical trials in systemic lupus erythematosus, rheumatoid arthritis, and multiple sclerosis are ongoing. It is important to note that anti-IFN-γ and anti-TNF-α mAb administration in the early phase of anti-MPO GN (day 20) had no effect on GN development, suggesting that this stage is driven by Th17-dominant immune responses. At this stage, innate cytokines such as IL-1β and IL-6 are likely critical in promoting Th17 differentiation and early immune activation. Schreiber et al. showed that IL-1 receptor blockade with anakinra protected against MPO antibody-induced necrotizing crescentic GN in mice (13). IL-6 has also been investigated in AAV, given its function in bridging innate and adaptive immune responses. Elevated serum IL-6 levels in AAV patients have been shown to decline with treatment and correlate with disease activity (14). Although these innate cytokine pathways were not the focus of this study, IL-1β and IL-6 are likely relevant during early disease and may be best examined in conjunction with Th17-targeted pathways in future investigations to fully delineate the cytokine networks driving disease initiation and progression.

Overall, our findings suggest that targeting downstream Th1 effector cytokines may be more effective in patients with established disease, where Th1 effector cells are already expanded and active within the kidney. This is particularly relevant for patients who present late in the disease course, when Th cell differentiation-targeting strategies may be less effective. By extending the treatment window and focusing on the effector phase, this study models a more clinically relevant scenario and provides mechanistic insight to support stage-specific therapeutic strategies in AAV. This work underscores the need for continued exploration of cytokine-targeted strategies in AAV and highlights the need to tailor interventions according to the dominant immune pathways active at different disease stages.

## Data Availability

The original contributions presented in the study are included in the article/**Supplementary Material**. Further inquiries can be directed to the corresponding author.

## Supplementary material

Supplementary Figure 1 The effect of IFN-γ blockade during Th17 dominant anti-MPO GN. In early establishing anti-MPO GN (day 20), treatment with anti-IFN-γ antibody at the timepoint of induced glomerulonephritis had no effect of glomerular injury [glomerular segmental necrosis and albuminuria **(A, B)**] and serum MPO-ANCA **(C)** compared to controls. MPO-specific delayed type hypersensitivity (DTH) was significantly reduced in mice receiving anti-IFN-γ antibody **(D)** as well as decreased MPO recall response for IFN-γ production from draining splenocytes **(E)**. No difference in splenic MPO recall responses for TNF-α and IL-17A was observed between groups **(F, G)**. *P<0.05.

Supplementary Figure 2 The effect of TNF-α blockade during early establish anti-MPO GN (day 20). Anti-MPO GN mice treated with anti-TNF-α antibody did not reduce glomerular segmental necrosis, albuminuria and serum MPO-ANCA compared to controls **(A-C)**. Blockade of TNF-α decreased MPO-specific delayed type hypersensitivity (DTH) footpad swelling **(D)**, increased serum concentration of TNF-α **(E)** and decreased anti-MPO recall response for TNF-α production from splenocytes **(F)**. No difference in MPO specific splenic recall responses of IFN-γ and IL-17A was observed between groups. *P<0.05, ***P<0.001, ****P<0.0001.
