# Supplementary material for: Neutralizing the Th1 effector cytokines, IFN-γ and TNF-α, attenuates established experimental autoimmune anti-myeloperoxidase glomerulonephritis
Source: Front Immunol. 2025 Jul 15;16:1589130. doi: 10.3389/fimmu.2025.1589130 (PMC12305698; doi:10.3389/fimmu.2025.1589130)
Supplement: Supplementary Figure 1 — The effect of IFN-γ blockade during Th17 dominant anti-MPO GN. In early establishing anti-MPO GN (day 20), treatment with anti-IFN-γ antibody at the timepoint of induced glomerulonephritis had no effect of glomerular injury [glomerular segmental necrosis and albuminuria (A, B)] and serum MPO-ANCA (C) compared to controls. MPO-specific delayed type hypersensitivity (DTH) was significantly reduced in mice receiving anti-IFN-γ antibody (D) as well as decreased MPO recall response for IFN-γ production from draining splenocytes (E). No difference in splenic MPO recall responses for TNF-α and IL-17A was observed between groups (F, G). *P<0.05. [file DataSheet1.docx]

**SUPPLEMENTARY FIGURE**

**
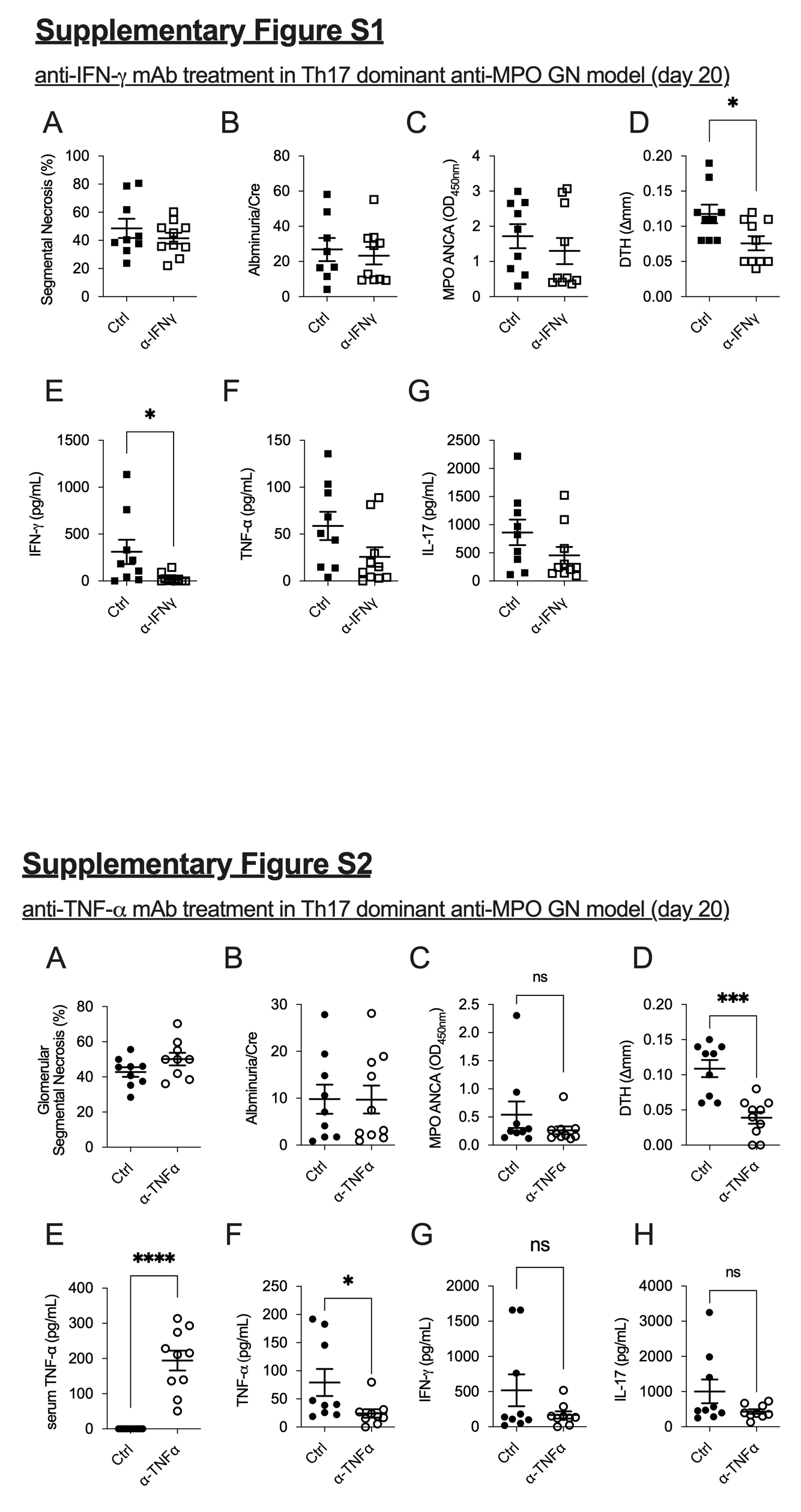
**

Supplementary Figure S1. The effect of IFN-γ blockade during Th17 dominant anti-MPO GN. In early establishing anti-MPO GN (day 20), treatment with anti-IFN-γ antibody at the timepoint of induced glomerulonephritis had no effect of glomerular injury (glomerular segmental necrosis and albuminuria [A-B]) and serum MPO-ANCA (C) compared to controls. MPO-specific delayed type hypersensitivity (DTH) was significantly reduced in mice receiving anti-IFN-γ antibody (D) as well as decreased MPO recall response for IFN-γ production from draining splenocytes (E). No difference in splenic MPO recall responses for TNF-α and IL-17A was observed between groups (F-G). *P<0.05

**
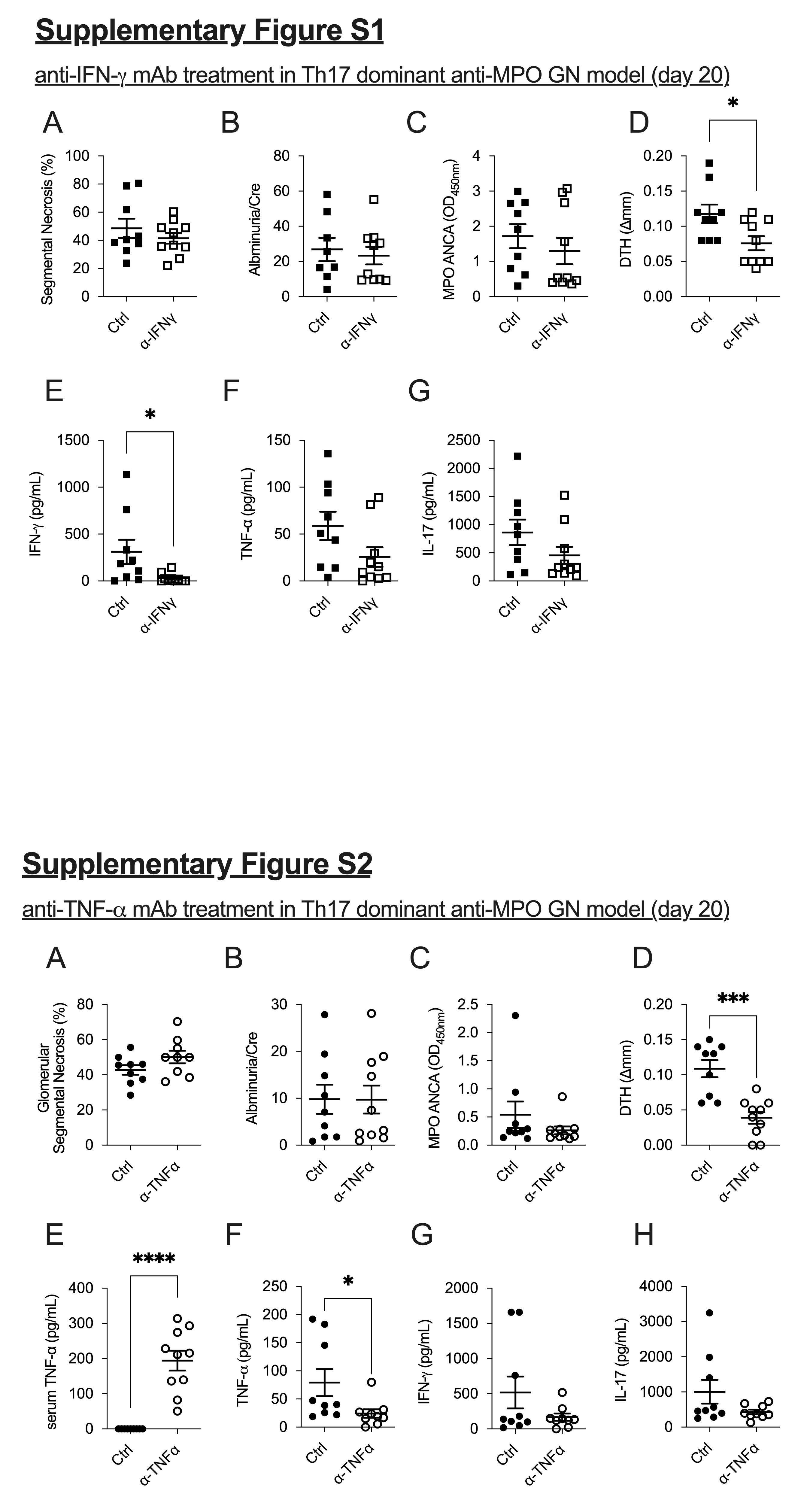
**

Supplementary Figure S2. The effect of TNF-α blockade during early establish anti-MPO GN (day 20). Anti-MPO GN mice treated with anti-TNF-α antibody did not reduce glomerular segmental necrosis, albuminuria and serum MPO-ANCA compared to controls (A-C). Blockade of TNF-α decreased MPO-specific delayed type hypersensitivity (DTH) footpad swelling (D), increased serum concentration of TNF-α (E) and decreased anti-MPO recall response for TNF-α production from splenocytes (F). No difference in MPO specific splenic recall responses of IFN-γ and IL-17A was observed between groups. *P<0.05, ***P<0.001, ****P<0.0001.
